# Recurrence in *Plasmodium vivax* malaria: a prospective cohort study with long follow-up from a coastal region in South-West India

**DOI:** 10.12688/f1000research.109577.2

**Published:** 2022-04-06

**Authors:** Divya Gandrala, Nitin Gupta, Alekhya Lavu, Vishnu Teja Nallapati, Vasudeva Guddattu, Kavitha Saravu

**Affiliations:** 1 Department of Medicine, Kasturba Medical College, Manipal, Manipal Academy of Higher Education, Manipal, Karnataka, 576104, India; 2 Department of Infectious Diseases, Kasturba Medical College, Manipal, Manipal Academy of Higher Education, Manipal, Karnataka, 576104, India; 3 Manipal Center for Infectious Diseases, Prasanna School of Public Health, Manipal Academy of Higher Education, Manipal, Karnataka, 576104, India; 4 Department of Pharmacy Practice, Manipal College of Pharmaceutical Sciences, Manipal Academy of Higher Education, Manipal, Karnataka, 576104, India; 5 Department of Data Science, Prasanna School of Public Health, Manipal Academy of Higher Education, Manipal, Karnataka, 576104, India

**Keywords:** Primaquine; relapse; severe malaria

## Abstract

**Background: ** India is endemic for *Plasmodium vivax* *(Pv) *malaria. Despite a decrease in incidence, its elimination is hampered by recurrences. This study aimed to characterize recurrences in *Pv* malaria and study its association with primaquine (PQ) usage.

**Methods:  **Symptomatic adult *Pv* patients were followed-up for up to 23 months for recurrences. The time to recurrence was compared by the PQ dosage they received using a log-rank test.

**Results: **Of the 294 malaria patients, 206 (70%) patients had *Pv* infection during the study period. A total of 20 (9.7%) recurrences were seen in 17 (8.2%) patients of *Pv*. The percentage of first-time recurrences were highest in the no PQ group (25%), followed by the weekly PQ group (20%), low dose daily PQ (8.2%) group, and high dose daily PQ group (3.1%).

**Conclusions: **Recurrence in *Pv* malaria is common, especially in those who receive an incorrect prescription of primaquine.

## Introduction

Malaria is a major global health problem, with around 228 million reported cases alone in 2018, most due to *Plasmodium falciparum* ( *Pf*). ^1^ Consequently, most reports on malaria concentrate on *Pf.* Traditionally, *Pf* has been described as the causative agent for severe malaria. However, recent reports have shown that malaria caused *by Plasmodium vivax* ( *Pv*) can also be severe. Although India represents a small percentage of the overall global malaria cases, it is responsible for nearly half of the total cases of *Pv.* ^2^^,^^3^ Despite a decline in the number of Malaria cases in India, the major roadblock to elimination is the tendency of *Pv* to relapse frequently, mainly when primaquine (PQ) is not prescribed or prescribed in sub-therapeutic dosage. ^4^^,^^5^ Therefore, the objective of the study was to calculate the incidence of recurrence in patients with *Pv* malaria and find the impact of PQ prescription practices on recurrence.

## Methods

A prospective observational study was conducted at Kasturba Hospital, Manipal in Udupi district of Karnataka State, India, for two years, from October 2016 to August 2018. The study was commenced after taking approval from the Institute's Ethical Committee (IEC 636/2016). All patients of either sex above 18 years of age who presented during the study period with fever and had *Pv* malarial parasites on the quantitative buffy coat (QBC) or peripheral smear examination were included in the study after taking written informed consent. Those patients with *Pf* or mixed infections ( *Pv* and *Pf*) were excluded. The article was reported according to the STROBE guidelines and all the criteria in the STROBE checklist were met. The sample size was calculated as 206 cases of *Pv*, considering recurrence prevalence as 31.5%, 95% level of confidence and 6.5% precision. ^6^

They diagnosis of *Pv* was based on the results of peripheral smear. A detailed history (including comorbidities), physical examination, and laboratory parameters were noted in a predefined case study form. In addition, the worst value of the variables during hospitalization was recorded. The patients were classified as having severe disease if they met the criteria for severity laid down by World Health Organisation (WHO). ^7^ Since the study aimed to record the prescription practices of treating physicians, the study objectives were not disclosed to them to avoid bias. The diagnosed cases were treated by the treating team. Glucose-6 Phosphate dehydrogenase (G6PD) levels were requested by the treating physician’s discretion. The enzyme activity was quantified by the manual spectrophotometric kinetic 'gold standard' method in the institutional biochemistry laboratory. G6PD deficiency was defined as less than 30% of mean G6PD activity.

Chloroquine was used in all patients for the treatment of *Pv* malaria. In an ideal situation, G6PD levels should be done prior to initiation of primaquine. If the levels are within normal range, WHO recommends 0.5 mg/kg primaquine to prevent relapse in tropical areas. The national guidelines in India, however, recommend 0.25 mg/kg according to their last available guidance. If the levels are low, weekly primaquine is recommended for 8 weeks. ^8^^,^^9^

The treating physicians decided the dosage of antimalarials, including PQ. The details of treatment, supportive care hospitalization days and mortality during hospital stay were noted. The primary outcome was microbiologically-confirmed recurrence at the end of the study period. Individuals were followed up telephonically every two months until the end of the study period for the development of fever recurrence. Additionally, individuals were asked to report if the fever recurred and were classified as recurrence if they were microscopically proven to have malaria again.

Statistical analysis was performed using Statistical Package for the Social Sciences version 23.0 **(SPSS, RRID:SCR_002865**, http://www-01.ibm.com/software/uk/analytics/spss/ **)**. Continuous variables were summarized as mean with standard deviation (SD) or median with interquartile range (IQR) (in skewed data). Categorical variables were summarized as the frequency with proportion. Overall, patients with *Pv* were divided into four groups according to PQ dosage- no PQ, weekly PQ, low dose daily PQ (0.25 mg/kg/day), and high dose daily PQ (0.5 mg/kg/day). The number of recurrences in each group were calculated. A Kaplan-Meier survival plot was generated to determine the survival function of recurrences according to PQ categories until 23 months' follow-up duration. Log-rank test was used to compare the survival function. A p-value of less than 0.01 was considered significant.

## Results

A total of 294 malaria cases were screened during the study period, of which 206 (70%) were *Pv,* 79 (27%) were *Pf*, and 9 (3%) were mixed ( *pv+pf*). A total of 29.6 % (87/294) cases had severe malaria. The proportion of severity, the requirement of supportive care, and mortality were comparable in both groups and summarized. The baseline clinical and laboratory features of patients with *Pv* and *Pf* malaria have been summarized in Table 1.

**Table 1.** Baseline clinical and laboratory features of patients with severe or non-severe vivax malaria.

	*Plasmodium vivax* (N=206)			*Plasmodium falciparum* (N=79)		
	Non-severe (n=144)	Severe (n=62)	P-value *	Non-severe (n=56)	Severe (n=23)	P-value *
**Age (years)**	36.1±14.2	40.6±14.1	0.76	34.4±14.6	38.59±13.1	0.41
Male gender	121(84%)	55 (88.5%)	0.38	48 (85.7%)	21 (95.5%)	0.22
Fever in days	4 (3,7)	4 (3,6)	0.83	4 (3,6)	6 (4,7)	0.01
Diabetes mellitus	15 (10.45%)	13 (21%)	0.04	5 (9%)	1 (4.3%)	0.48
Hypertension	14 (9.72%)	10 (16.1%)	0.18	5 (9%)	3 (13.04%)	0.58
Pulse rate (beats/min)	88±14	92±16	0.22	88±11	87±12	0.47
Respiratory rate (breaths/min)	19±2	20±5	0.007	18±2	21±6	0.001
ARDS	5 (3.5%)	0	0.001	3 (5.4%)	0	0.005
Systolic blood pressure (mmHg)	120±14	114±20	0.001	121±17	113±15	0.8
Diastolic blood pressure (mmHg)	77±8	73±12	0.002	77±9	73±13	0.15
Shock	7 (3.4%)	0	<0.001	4 (7.1%)	0	0.001
Pallor	5 (3.5%)	6 (9.8%)	0.07	5 (8.9%)	4 (18.2%)	0.28
Icterus	44 (30.6%)	0	<0.001	17 (30.4%)	0	<0.001
Impaired consciousness	3 (2.1%)	0	0.009	1 (1.8%)	0	0.108
Convulsion	1 (0.7%)	0	0.136	1 (1.8%)	0	0.108
Metabolic acidosis	3 (2.1%)	0	0.010	1 (1.8%)	0	0.108
Renal failure	10 (6.9%)	0	<0.001	3 (5.4%)	0	0.005
Splenomegaly	17 (11.8%)	14 (23%)	0.04	11 (19.6%)	8 (36.4%)	0.15
Hepatomegaly	8 (5.6%)	15 (24.6%)	<0.001	6 (10.7%)	4 (18.2%)	0.41
Hemoglobin (g/dL)	13.4 ± 1.9	12.8 ± 2.5	0.01	12.9 ± 2.1	12.2 ± 3.2	0.03
Hematocrit (%)	39.7 ± 5.6	37.8 ± 7.3	0.02	38 ± 6.5	35.7 ± 9	0.12
Total Leukocyte count (cells/mm ^3^)	5655 ± 2154	5813 ± 2978	0.008	5049 ± 1804	7632 ± 4516	0.001
Platelet count (cells/mm ^3^)	74500 (49250,113250)	47000 (30750,79500)	0.001	75000 (48500,136250)	39000 (16000,96500)	0.007
Plasma Glucose (mg/dL)	132±54	149±60.1	0.13	139±70.8	132±44.5	0.15
Blood Urea (mg/dL)	25 (20,31)	32 (23,45.5)	<0.001	24 (19, 30)	32 (22, 65)	0.01
Serum Creatinine (mg/dL)	0.98±0.27	1.17±0.48	<0.001	1.01±0.42	1.7±2.01	0.01
Total Bilirubin (mg/dL)	1.49 ± 0.62	3.8 ± 2.9	<0.001	1.5 ± 0.6	6.8 ± 7.56	<0.001
Direct Bilirubin (mg/dL)	0.6 ± 0.3	2.08 ± 2.42	<0.001	0.6 ± 0.4	4.23 ± 5.16	<0.001
Aspartate transaminase (IU/L)	33.5 (24,43)	49 (30,65.5)	<0.001	36 (25, 58.5)	47.5 (37.3, 96)	0.02
Alanine transaminase (IU/L)	34 (22,53)	43.5 (27.2,87.7)	0.01	43 (24, 70)	54.5 (31.7, 103.2)	0.2
Alkaline phosphatase (IU/L)	75 (60,94)	99 (76.3,144.7)	<0.001	93 (61, 115.8)	122.5 (76.3, 181.5)	0.02

* Categorical variables are summarized as the frequency with proportion whereas continuous variables are summarized as either mean (±SD) or median (IQR). Chi-square or Fischer's exact test and Independent sample *t*-test or Mann Whitney *U* test were performed, *p*-value less than 0.05 shows the statistically significant difference and shown in bold font. ARDS: Acute Respiratory Distress Syndrome.

Of 206 *Pv* cases included in the study, there were 20 recurrences in 17 (8.5%) patients. The median time to follow-up was 388 (293–567) days. The median time to the first recurrence was 83 (66.5–242.5) days.

**Figure 1.**
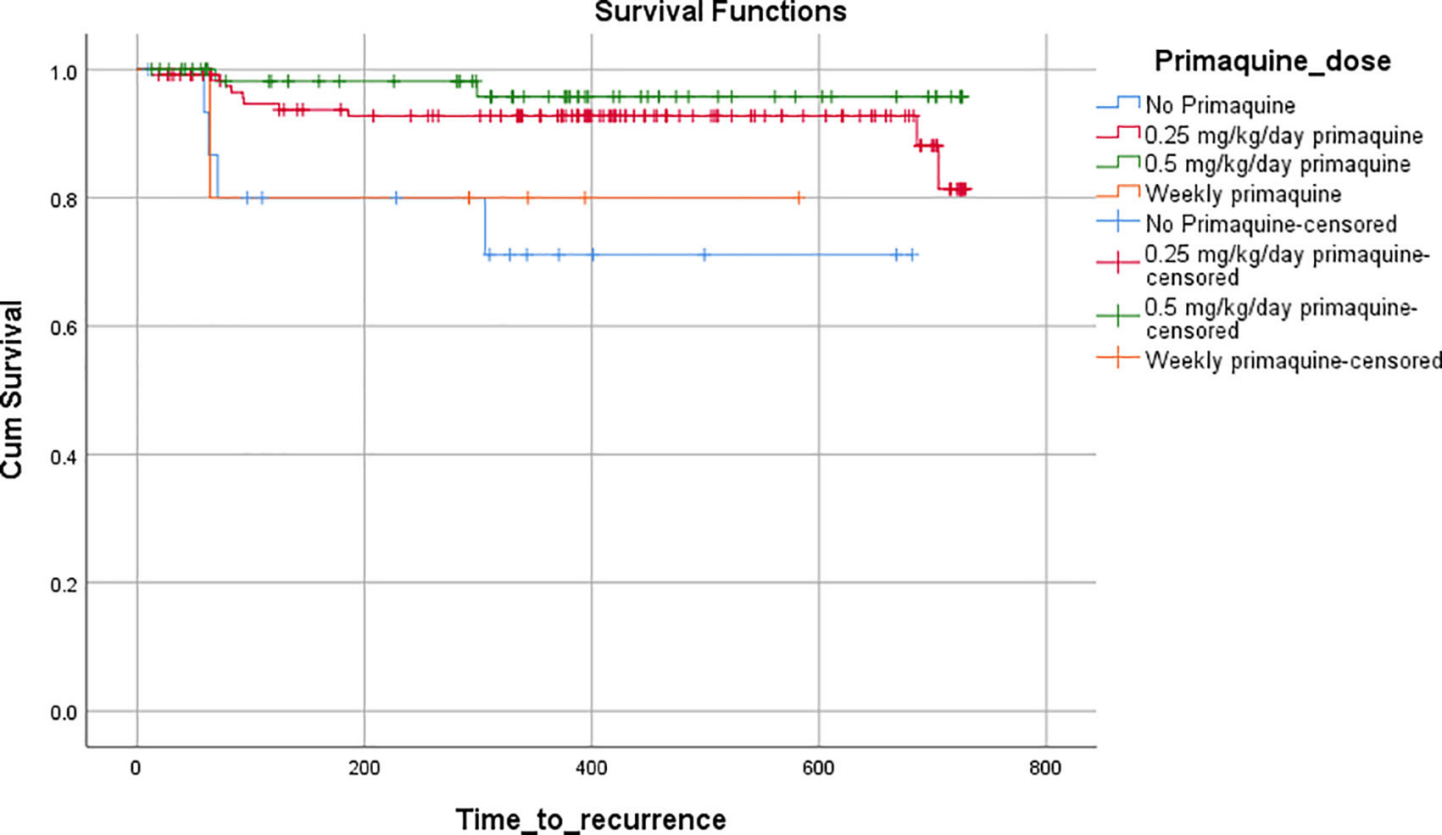
Time to recurrence stratified according to primaquine dosing on Kaplan-Meier survival analysis.

Of the 206 patients with *Pv*, G6PD levels could be done in 196 patients only, out of which nine patients were found to have low G6PD levels ( Table 2). No case of PQ-induced hemolysis was noted in our cohort. The dose of PQ was significantly associated with recurrences on the Chi-square test (p<0.001). The percentage of first-time recurrences were highest in the no PQ group (25%), followed by the weekly PQ group (20%), low dose daily PQ (8.2%) group, and high dose daily PQ group (3.1%) ( Table 2). A Kaplan-Meier curve was plotted to compare the median time to recurrence in each of the PQ-based groups, and the difference was found to be significant on the log-rank test (p=0.009) ( Figure 1).

**Table 2.** Recurrences in *Plasmodium vivax* cases stratified according to G6PD levels and primaquine prescription patterns.

Primaquine (PQ)	G6PD levels low (n=9)		G6PD levels normal (n=187)		G6PD not done (n=10)	
PQ dose	Total prescribed	Recurrences	Total prescribed	Recurrences	Total prescribed	Recurrences
No PQ	1	0	10	2	5	2
Weekly PQ	5	1	0	0	0	0
Daily PQ (0.25 mg/kg)	3	1	114	8	4	1
Daily PQ (0.5 mg/kg)	0	0	63	2	1	0

PQ: Primaquine; G6PD: Glucose 6 Phosphate dehydrogenase.

## Discussion

Udupi district has a population of 1,177,908 with an area of 3,582 sq. km and is located 13°32′ 24.43′′ N latitude and 74°52′26.78′′ E longitude, with typical tropical climatic conditions. The monsoon in this region starts in June and extends till October, with an average rainfall of more than 4000mm every year. The catchment area of our hospital encompasses both the rural and urban populations of coastal and interior Karnataka, Goa and Kerala. *Pv* is the largest infecting species in this region, followed by *Pf.* ^10^^,^^11^ The same trend is noted in other parts of India. ^12^^,^^13^

As expected, all but one recurrence were seen in patients with *Pv.* The percentage recurrence in *Pv* cases was close to 10%, which was considerably lower than recurrences reported in the previous series (24–38%). ^14^^,^^15^ Like a previous study, all recurrent cases had mild symptoms, presumably due to the development of acquired immunity from the previous episode. ^16^ The median time to recurrence was 83 days in our study, similar to previously published studies. ^14^ Those patients for whom PQ was not used had higher rates of recurrence.

We classified the patients according to the G6PD levels because in those patients where G6PD levels were not done, we couldn’t judge the correctness of the prescription choices. The idea was to show that a G6PD levels were not even offered to some patients. On top of that, many patients were given incorrect prescriptions despite G6PD levels indicating otherwise. Of the 16 patients for whom no PQ was used, only one patient had proven low levels of G6PD. This implies that PQ was not prescribed because of possible lack of awareness. This reflects the need to reinforce the fact that G6PD levels should be done in all patients with *Pv* and the prescriptions should be guided by the G6PD levels.

Since the recurrence rates were lowest in those wher primaquine was used as 0.5 mg/kg , patients with normal G6PD levels should receive 0.5 mg/kg prophylaxis. Even with a lower dose of PQ (0.25 mg/kg), the recurrences are lower when compared to those who were not given PQ. Similar results were observed in other studies as well. ^17^ Since the study was done in a tertiary care hospital where G6PD levels and specialist referrals are available, the study cannot be generalized to primary care settings. Similar widespread prescription audits are required all over the country to understand the practices and pattern of recurrences in patients with *Pv.*

### Limitations of the study

Self-limiting intermittent recurrences that are asymptomatic could not be ruled out as symptom-based screening for recurrence was done. The genotyping of recurrences could not be done to discern relapse and reinfection. The possibility of non-compliance cannot be ruled out as PQ therapy was unsupervised. New *Pv* infections could be differentiated from relapses in this study. The study follow-up was of long duration so recurrences at 1-2 years may or may not be related to the PQ dose.

## Conclusions

The study concludes that, *Pv* may be associated with recurrences, especially when PQ prescription practices are not aligned with international evidence based recommendations. G6PD levels should be ascertained in all patients with *Pv* malaria, and daily PQ prophylaxis should be given to those patients with normal G6PD levels. There is a need for improving prescription practices amongst primary care physicians through regular educational interventions.

## Data availability

Data cannot be shared due to ethical and security concerns, however a de-identified dataset with all the details can be shared with reviewer or readers at reasonable request to corresponding author.

## Author's contributions

All authors have read and approved the final manuscript. The requirements for authorship have been met, and each author believes that the manuscript represents honest work.

Divya Gandrala: Conceptualization, Data curation, Formal analysis, Investigation, Methodology, Project administration, Writing-original draft preparation, Writing-Review & Editing

Nitin Gupta: Formal analysis, Validation, Writing-original draft preparation, Writing-Review & Editing

Alekhya Lavu: Data curation, Formal analysis, Investigation, Methodology

Vishnu Teja Nallapati: Writing-original draft preparation, Writing-Review & Editing

Vasudeva Guddattu: Formal analysis, Software, Writing-original draft preparation, Writing-Review & Editing

Kavitha Saravu: Conceptualization, Data curation, Formal analysis, Project administration, Supervision, Validation, Writing-original draft preparation, Writing-Review & Editing
