# Supplementary material for: Recurrence in Plasmodium vivax malaria: a prospective cohort study with long follow-up from a coastal region in South-West India
Source: F1000Res. 2022 Apr 6;11:279. Originally published 2022 Mar 4. [Version 2] doi: 10.12688/f1000research.109577.2 (PMC9005986; doi:10.12688/f1000research.109577.2)
Supplement: Peer review history

# Peer review: Reviewer response for version 2

**DOI:** 10.5256/f1000research.123067.r129946

**Published:** 2022-04-12

**Authors:** Cindy Chu

**Affiliations:** 1 Shoklo Malaria Research Unit, Mahidol-Oxford Tropical Medicine Research Unit, Faculty of Tropical Medicine, Mahidol University, Mae Sot, Tak Province, Thailand

The authors have improved the clarity of the manuscript. The discussion and conclusion are consistent with the study methods and results. The conclusion is clear.

Is the work clearly and accurately presented and does it cite the current literature?

Partly

If applicable, is the statistical analysis and its interpretation appropriate?

Partly

Are all the source data underlying the results available to ensure full reproducibility?

No

Is the study design appropriate and is the work technically sound?

Partly

Are the conclusions drawn adequately supported by the results?

Partly

Are sufficient details of methods and analysis provided to allow replication by others?

No

Reviewer Expertise:

Plasmodium vivax, radical cure with 8-aminoquinolines, G6PD deficiency

I confirm that I have read this submission and believe that I have an appropriate level of expertise to confirm that it is of an acceptable scientific standard.

# Peer review: Reviewer response for version 1

**DOI:** 10.5256/f1000research.121091.r126315

**Published:** 2022-03-18

**Authors:** Cindy Chu

**Affiliations:** 1 Shoklo Malaria Research Unit, Mahidol-Oxford Tropical Medicine Research Unit, Faculty of Tropical Medicine, Mahidol University, Mae Sot, Tak Province, Thailand

This manuscript describes the prevalence and some epidemiologic aspects of *Plasmodium vivax (Pv)*, *Plasmodium falciparum (Pf)*, and mixed-species infection in Southwest India. The study is a prospective observational study and appears to be part of a prescription audit. There is a sub-analysis that compares the recurrence rates of *Pv *malaria among four groups: no primaquine, weekly primaquine, low dose primaquine, and high dose primaquine. Primaquine (PQ) was not supervised. The data are useful, however, the presentation of the study design, sample size, and methods need more explanation and some of the results need more precision so that the correct conclusions can be made.

 ABSTRACT:

- The conclusion states that recurrence in *Pv* is common especially when PQ prescription is inappropriate. I suggest using a more precise word instead of ‘inappropriate’.

INTRODUCTION:

- The last sentence of the introduction states that the objective of the study was to calculate the incidence of recurrence in patients with *Pv* malaria and find the impact of PQ prescription practices on recurrence. The authors then conduct a prospective observational study. However, this analysis appears to be a part of a prescription audit (as stated in the Discussion) rather than a study specifically designed to compare drug groups. This becomes more apparent in the study methods where it is stated: “the study aimed to record the prescription practices of treating physicians”. Moreover, the inclusion of *Pf* and mixed cases does not match the study objective or the sample size calculation. Please specify the study design and study outcome(s).

METHODS:

- In the methods section, it is stated that the treating physicians decided on the dosage of antimalarials. Since PQ is being used, can the authors describe how G6PD testing is used before prescribing PQ? Which schizonticidal treatments are prescribed?

RESULTS:

- In the results section, 294 malaria cases were enrolled. The sample size is specified to be 206. Do the authors mean that 294 malaria cases were screened?
- Figure 1 describes the data by malaria species but the analysis is by drug group. Please modify so that the trial diagram is consistent with the data analysis.
- The chi-squared test would normally be used to analyse 2x2 groups. The chi-squared test as used in the results section is for 2x4 groups ( *Pv* yes/no and 4 different drug regimens). The Kaplan Meier figure is the correct analytic tool so the chi-squared result could be removed.

DISCUSSION:

- There is one paragraph dedicated to severe malaria, however, to this point in the manuscript, the outcome of the study was *Pv* recurrence. This paragraph (the *Pf *part) does not seem related the stated study outcome. Please clarify.
- What is the relevance of G6PD activity levels in this study for *Pv* recurrence? I understand the G6PD test would be used to determine which PQ regimen should be prescribed, but that has not yet been described in the manuscript and it should be added. There is a statement that only one patient in the no PQ group had low G6PD levels. Presumably any participant with low G6PD activity should have been in the no PQ or weekly PQ groups. Please explain.
- The following sentence “This reflects the need to reinforce the importance of primaquine prescription in patients with *Pv*”, needs more specific language. Do the authors mean low G6PD levels reflect the need to reinforce the importance of PQ prescription? Or the need for G6PD testing before prescribing PQ?
- The following sentence “The rates were lower in the daily PQ group even when they were used at a lower dose”, needs more specific language. What rates were lower? What is 'they' - the patients or PQ?

STUDY LIMITATIONS:

- New *Pv* infections cannot be differentiated from relapses in this study. The study follow-up has a very long duration so recurrences at 1-2 years may or may not be related to the PQ dose. This should be included in study limitations and appropriate citations included.

CONCLUSION:

- What do the authors mean by inappropriate PQ prescription? How do they determine whether recurrences are associated with inappropriate prescription when new infections cannot be differentiated from relapses or if results are affected by non-compliance? What prescription practices need to be improved? It seems that if the study design and methods are clearer, then the results and conclusion will be better supported.

Is the work clearly and accurately presented and does it cite the current literature?

Partly

If applicable, is the statistical analysis and its interpretation appropriate?

Partly

Are all the source data underlying the results available to ensure full reproducibility?

No

Is the study design appropriate and is the work technically sound?

Partly

Are the conclusions drawn adequately supported by the results?

Partly

Are sufficient details of methods and analysis provided to allow replication by others?

No

Reviewer Expertise:

Plasmodium vivax, radical cure with 8-aminoquinolines, G6PD deficiency

I confirm that I have read this submission and believe that I have an appropriate level of expertise to confirm that it is of an acceptable scientific standard, however I have significant reservations, as outlined above.

# Response

**Published:** 2022-03-31

**Authors:** Kavitha Saravu

**Affiliations:** Kasturba Medical College, India

**This manuscript describes the prevalence and some epidemiologic aspects of *Plasmodium vivax (Pv)*, *Plasmodium falciparum (Pf)*, and mixed-species infection in Southwest India. The study is a prospective observational study and appears to be part of a prescription audit. There is a sub-analysis that compares the recurrence rates of *Pv *malaria among four groups: no primaquine, weekly primaquine, low dose primaquine, and high dose primaquine. Primaquine (PQ) was not supervised. The data are useful, however, the presentation of the study design, sample size, and methods need more explanation and some of the results need more precision so that the correct conclusions can be made.**

**Reply:** We appreciate the constructive suggestions by the reviewer. We have modified our manuscript as per the suggestions. A point-wise reply, explanation, and modification are as follows.

**ABSTRACT:**

- **The conclusion states that recurrence in *Pv* is common especially when PQ prescription is inappropriate. I suggest using a more precise word instead of ‘inappropriate’.**

**Reply:** The inappropriate word was changed to ‘Incorrect’.

**INTRODUCTION:**

- **The last sentence of the introduction states that the objective of the study was to calculate the incidence of recurrence in patients with *Pv* malaria and find the impact of PQ prescription practices on recurrence. The authors then conduct a prospective observational study. However, this analysis appears to be a part of a prescription audit (as stated in the Discussion) rather than a study specifically designed to compare drug groups. This becomes more apparent in the study methods where it is stated: “the study aimed to record the prescription practices of treating physicians”. Moreover, the inclusion of *Pf* and mixed cases does not match the study objective or the sample size calculation. Please specify the study design and study outcome(s).**

**Reply: **The study was designed as a prospective observational study where patients with malaria were enrolled at baseline. A follow-up component was added to estimate the percentage of patients with recurrence. Prescription audit was a main component of the study which is a type of observational study. Since there is a follow-up component built in the study to estimate the percentage of recurrences, we described our study as a prospective observational study.

 We included all malaria cases but the analysis was restricted to Pv as recurrences in Pf are not common. The primary objective was to study the recurrences in Pv. The sample size was also calculated based on that primary objective. Baseline parameters were collected at presentation and prescription patterns were recorded. The patients were followed up for recurrences. The inclusion of other cases was shown for the sake of comprehensiveness but as the reviewer suggests, they appear to be distractors. We have therefore removed all data on Pf and mixed malaria from this study.

**METHODS:**

- **In the methods section, it is stated that the treating physicians decided on the dosage of antimalarials. Since PQ is being used, can the authors describe how G6PD testing is used before prescribing PQ? Which schizonticidal treatments are prescribed?**

**Reply:** We appreciate the suggestion and have incorporated the following statement, “Chloroquine was used in all patients for the treatment of Pv malaria. In an ideal situation, G6PD levels should be done prior to initiation of primaquine. If the levels are within normal range, WHO recommends 0.5 mg/kg primaquine to prevent relapse in tropical areas. The national guidelines in India, however, recommend 0.25 mg/kg according to their last available guidance. If the levels are low, weekly primaquine is recommended for 8 weeks.”

**RESULTS:**

- **In the results section, 294 malaria cases were enrolled. The sample size is specified to be 206. Do the authors mean that 294 malaria cases were screened?**

**Reply:** We enrolled 294 cases with 206 being Pv. For the other patients who had Pf or mixed, the baseline parameters were recorded. However, as mentioned earlier, we have removed the data on Pf and mixed malaria for clarity as per the reviewer’s suggestion.

  

- **Figure 1 describes the data by malaria species, but the analysis is by drug group. Please modify so that the trial diagram is consistent with the data analysis.**

**Reply:** The table shows the trial diagram in a more succinct manner so Figure 1 is removed to avoid confusion. Since we have removed Pf and mixed malaria, Figure 1 seemed redundant anyways.

  

- **The chi-squared test would normally be used to analyse 2x2 groups. The chi-squared test as used in the results section is for 2x4 groups ( *Pv* yes/no and 4 different drug regimens). The Kaplan Meier figure is the correct analytic tool so the chi-squared result could be removed.**

**Reply: **Chi-square test was meant for comparing severe and non-severe malaria at baseline. However, we have removed that from the main table as suggested.

**DISCUSSION:**

- **There is one paragraph dedicated to severe malaria, however, to this point in the manuscript, the outcome of the study was *Pv* recurrence. This paragraph (the *Pf *part) does not seem related the stated study outcome. Please clarify.**

**Reply:** Since we classified the baseline features into severe and non-severe, we had discussed this in the discussion section. The paragraph has now been removed as suggested.

  

- **What is the relevance of G6PD activity levels in this study for *Pv* recurrence? I understand the G6PD test would be used to determine which PQ regimen should be prescribed, but that has not yet been described in the manuscript and it should be added. There is a statement that only one patient in the no PQ group had low G6PD levels. Presumably, any participant with low G6PD activity should have been in the no PQ or weekly PQ groups. Please explain.**

**Reply:** We agree with the suggestion and have added the following statement. “If the levels are within normal range, WHO recommends 0.5 mg/kg primaquine to prevent relapse in tropical areas. The national guidelines in India, however, recommend 0.25 mg/kg according to their last available guidance. If the levels are low, weekly primaquine is recommended for 8 weeks.”

 We classified the patients according to the G6PD levels because, in those patients where G6PD levels were not done, we couldn’t judge the correctness of the prescription choices. The idea was to show that G6PD levels were not even offered to some patients. On top of that, many patients were given incorrect prescriptions despite G6PD levels indicating otherwise. Since the treatment plans were not decided by the study team and the objective of the study was to study the correctness or incorrectness of the prescription, we couldn’t presume that the participant with low G6PD activity would be in the no PQ group or weekly PQ group. Therefore, we mentioned in the statement that only one patient in the no PQ group had low G6PD levels. PQ prescription pattern in patients with low G6PD has been depicted in Table 2.

  

- **The following sentence “This reflects the need to reinforce the importance of primaquine prescription in patients with *Pv*”, needs more specific language. Do the authors mean low G6PD levels reflect the need to reinforce the importance of PQ prescription? Or the need for G6PD testing before prescribing PQ?**

**Reply:** We meant that G6PD levels should be done in all patients and the prescriptions should be guided by the G6PD levels. Those with low levels should be given weekly prescriptions and those with normal levels should receive 0.5 mg/kg prophylaxis.

  

- **The following sentence “The rates were lower in the daily PQ group even when they were used at a lower dose”, needs more specific language. What rates were lower? What is 'they' - the patients or PQ?**

**Reply:** The recurrence rates were lower in the daily PQ group even when the PQ was used at a lower dose i.e 0.25 mg/kg.

**STUDY LIMITATIONS:**

- **New *Pv* infections cannot be differentiated from relapses in this study.** **The study follow-up has a very long duration so recurrences at 1-2 years may or may not be related to the PQ dose.** **This should be included in study limitations and appropriate citations included**.

**Reply:** We agree with the suggestion. The same has been added.

**CONCLUSION:**

- **What do the authors mean by inappropriate PQ prescription? How do they determine whether recurrences are associated with inappropriate prescription when new infections cannot be differentiated from relapses or if results are affected by non-compliance? What prescription practices need to be improved? It seems that if the study design and methods are clearer, then the results and conclusion will be better supported.**

**Reply:** We have revised the paper so that the conclusion makes more sense. The meaning of inappropriate prescription practices has been described in the discussion section. Examples included, not giving PQ or giving them at a lower dosage. The significant log-rank test between the groups suggests that poor prescription practices may have some role. Prescription practices are required to be aligned with the current evidence-based recommendations.

# Peer review: Reviewer response for version 1

**DOI:** 10.5256/f1000research.121091.r126318

**Published:** 2022-03-16

**Authors:** Vettakkara Kandy Muhammed Niyas

**Affiliations:** 1 Department of Infectious Diseases, Kerala Institute of Medical Sciences, Thiruvananthapuram, Kerala, India

Gandrala *et al.* has performed a prospective cohort study in which malaria patients were followed up for a median duration of 388 days. There were 20 cases (9.7%) of recurrences in 206 cases of *P. vivax* malaria, occurring at a median time of 83 days. The authors observed that recurrences were highest in the no primaquine (PQ) group and were least in the high dose daily PQ group. The authors also use a Kaplan-Mier analysis to show that there was a significant difference in the median time to recurrence among various PQ-based groups. However, the median time to recurrence in each of these groups is not mentioned.

 The authors mention that no PQ-related haemolysis occurred in the cohort. However, it may be noted that no patient with low G6PD levels received a high dose of daily primaquine (0.5mg/kg). It is interesting to note that despite normal G6PD levels majority of patients did not receive a high dose PQ regimen. This data calls for awareness programmes among physicians regarding the optimal dosing of primaquine to prevent relapse. The authors rightly conclude that the results of the study cannot be generalised to primary care settings due to limited access to G6PD levels.

Is the work clearly and accurately presented and does it cite the current literature?

Yes

If applicable, is the statistical analysis and its interpretation appropriate?

Yes

Are all the source data underlying the results available to ensure full reproducibility?

Yes

Is the study design appropriate and is the work technically sound?

Yes

Are the conclusions drawn adequately supported by the results?

Yes

Are sufficient details of methods and analysis provided to allow replication by others?

Yes

Reviewer Expertise:

Infectious Diseases, Tropical Infections

I confirm that I have read this submission and believe that I have an appropriate level of expertise to confirm that it is of an acceptable scientific standard.
